# Sulphate-reducing bacteria-mediated pyrite formation in the Dachang Tongkeng tin polymetallic deposit, Guangxi, China

**DOI:** 10.1038/s41598-023-38827-x

**Published:** 2023-07-19

**Authors:** Fuju Jia, Xiangtong Lei, Yongfeng Yan, Yaru Su, Hongjun Zhou, Honglian Wei, Yuan Yuan, Chao Zou, Xianwen Shi, Ceting Yang

**Affiliations:** 1 Department of Earth Sciences, Kunming University of Science and Technology, Kunming, 650093 Yunnan China; 2 Yunnan Key Laboratory for Paleobiology & MEC International Joint Laboratory for Paleobiology and Paleoenvironment, Institute of Paleontology, Yunnan University, Kunming, 650500 China; 3 Guangxi 215 Geological Team Co., Ltd., Liuzhou, 545006 Guangxi China; 4 Guangxi China-Tin Group Tongkeng Co., Ltd., Nandan, 547205 Guangxi China

**Keywords:** Geochemistry, Mineralogy, Marine microbiology

## Abstract

Mediation by sulphate-reducing bacteria (SRB) is responsible for pyrite (FeS_2_) formation. The origin of the Dachang tin polymetallic ore field is related to the mineralisation of submarine hydrothermal vent sediments. Here, we investigated SRB in these ores via morphological, chemical, and isotopic analyses. Polarised and scanning electron microscopy indicated that trace SRB fossils in the metal sulphide ore were present in the form of tubular, beaded, and coccoidal bodies comprising FeS_2_ and were enclosed within a pyrrhotite (FeS) matrix in the vicinity of micro-hydrothermal vents. The carbon (C), nitrogen (N), and oxygen (O) contents in the FeS_2_ synthesised by SRB were high, and a clear biological Raman signal was detected. No such signals were discerned in the peripheral FeS. This co-occurrence of FeS, FeS_2_, and the remains of bacteria (probably chemoautotrophic bacteria) was interpreted as the coprecipitation process of SRB-mediated FeS_2_ formation, which has, to the best of our knowledge, not been reported before. Our study also illustrates that combined energy-dispersive X-ray spectroscopy, Raman spectroscopy, and isotopic analysis can be used as a novel methodology to document microbial-mediated processes of mineral deposition in submarine hydrothermal vent ecology on geological time scales.

## Introduction

Submarine hydrothermal vents are characterised by original materials and environmental conditions hypothesised to be required for abiogenesis and are considered a potential location for the origin of life on Earth^1–3^. Modern submarine hydrothermal vents often exhibit vibrant biological assemblies^4–6^. Microbes (e.g., chemoautotrophic bacteria) can acquire the materials and energy needed for biotic activities from hydrothermal vent fluids; other organisms (e.g., tubular worms, bivalves, and arthropods) directly feed on, or co-exist with, microbes, and together constitute submarine hydrothermal vent ecosystems^5^. Submarine hydrothermal vent systems, in addition to their lush animal communities^6^, are also sites for the enrichment of polymetallic sulphide deposits^7,8^. Because submarine vents are often connected to the deep part of the ocean crust through faults, hot water circulation constantly extracts metal substances from the crust, and metal ions migrate with the hydrothermal fluid to the seabed where they are precipitated. Magnetite, pyrite (FeS_2_), chalcopyrite, galena, sphalerite, and other minerals are commonly found in the deposits of submarine hydrothermal vents^9,10^.

FeS_2_ has stable chemical properties in reduced sediments and represents the most abundant sulphide in submarine hydrothermal vent systems^11,12^. Although the mechanism of sedimentary FeS_2_ formation is still a matter of debate, experiments have demonstrated that microorganisms in sediments play a crucial role in the process of sedimentary FeS_2_ formation^13^. To clarify the mechanism of FeS_2_ synthesis by microbes, numerous scientific experiments have attempted to simulate in-vivo conditions in a laboratory environment to microbially synthesise FeS_2_. In previous experiments on biomineralisation by sulphate (SO_4_^2−^)-reducing bacteria (SRB), the final product yielded only mackinawite and greigite, not FeS_2_, even with the addition of aqueous iron (Fe) or Fe minerals^14–20^. A breakthrough was not achieved until recent experiments succeeded in forming FeS_2_ spheroids in FeS-containing biofilms produced by SRB in the presence of organic compounds^21–24^. Accordingly, FeS_2_ is widely suspected to contain traces of biological activity^21^; however, only a few examples of the preservation of microfossils^25–30^ have been noted, with no documents on the SRB**-**mediated formation of FeS_2_.

The origin of the Dachang tin polymetallic ore field in Guangxi is considered to be related to the mineralisation of submarine vent sediments in the late Devonian^31–33^. In addition, recent research has shown that the ore-bearing rocks are reef limestones rich in fossils and other organic components^33^. This tentatively indicates the presence of microbial traces in the Dachang tin polymetallic sulphide ore. In this study, suspected traces of SRB in polymetallic sulphide samples and compositional data were analysed using a polarising microscope and scanning electron microscopy with energy-dispersive X-ray spectroscopy (SEM‒EDX). We also used Raman spectroscopy to detect biological spectral signals and analyse the stable isotope compositions of Fe, S, C, and O in SRB-related minerals. We thus infer the living environment of SRB and the potential processes of SRB-mediated FeS_2_ formation in Devonian submarine hydrothermal vents systems.

## Geological setting

The Dachang tin polymetallic field is a world-renowned non-ferrous metal production area, with large proven resources of tin (Sn; 1.47 million tonnes), Zn (6.80 million tonnes), Pb (1.76 million tonnes), Sb (1.38 million tonnes), and Cu (0.37 million tonnes), as well as associated economically viable and rare elements such as indium (In), cadmium (Cd), and gallium (Ga)^9,10^. The ore-bearing strata of the deposit are Devonian limestone, reefal limestone, siliceous rock, and shale; exposed Late Cretaceous granite porphyry and diorite porphyrite veins exist in the mining area as well. It has been suggested that the deposit is of hydrothermal or composite origin, derived from Late Cretaceous granite^34–36^; however, other researchers reported that the formation of the deposit was related to the mineralisation of submarine vent sediments^31–33^.

### Regional geology

**Figure 1:**
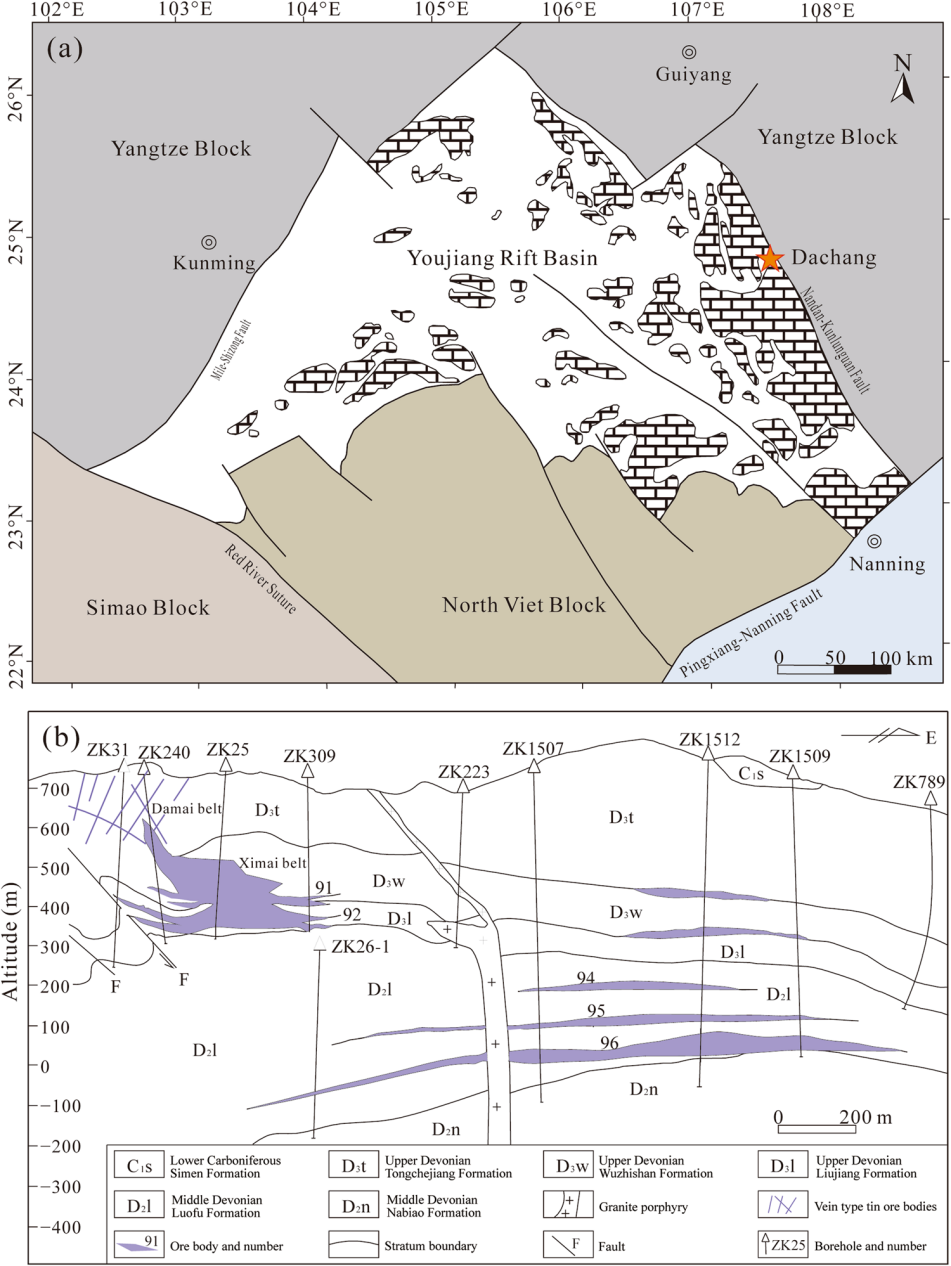
Geotectonic location and ore deposit profile. (**a**) Geotectonic location of the Dachang Sn polymetallic ore field in Guangxi (Modified from ref.^40^). (**b**) Geological profile of the Tongkeng Sn polymetallic deposit (Modified from ref.^41^). This map was created using Adobe Illustrator 2020.

The Youjiang Basin, located on the southwest edge of the Yangtze Craton in southwest China, was formed by composite parts of several tectonic units, including the Yangtze, North Vietnam, and Simao blocks. The Dachang Sn polymetallic ore field in Guangxi is located in the far northeast of the Youjiang Basin (Fig. 1a). The Youjiang Basin evolved in two stages: the Hercynian (Devonian‒Permian) saw a passive continental margin rift stage, and the Indosinian (Early‒Middle Triassic) saw a back-arc rift basin stage. In the Devonian, a NW‒SE faulted sub-basin was formed inside the Youjiang Basin, in which the southwest margin of the Yangtze Craton underwent extensional faulting^37^. In the early Middle Devonian (Nabiao Formation period), reefs developed in the Dachang area with local underwater uplifts. Synsedimentary faults developed in the Dachang area from the Late Devonian Liujiang to Wuzhishan periods, and siliceous rocks and banded limestone were widely deposited^31^. Some studies have shown that there were two stages of submarine hydrothermal vent sedimentation and mineralisation in the Late Devonian Dachang Sn polymetallic ore field. In the first stage, the Yanjiang Formation strata (D3l; Fig. 1b), dominated by banded siliceous rocks, were formed, and the No. 92 cassiterite-sulphide type orebody was produced. In the second stage, the Wuzhishan Formation strata (D3w; Fig. 1b), primarily composed of banded marble and siliceous rocks, was formed, and the No. 91 cassiterite-sulphide type orebody was produced^31,38,39^.

### Ore deposit geology

The ore-bearing strata are dominated by sandstone, shale, and carbonate rocks, with local carbonaceous mudstone and siliceous rocks. The ore field is primarily composed of five Sn polymetallic deposits, namely Tongkeng (Sn‒Zn‒Pb), Gaofeng (Sn‒Zn‒Pb), Dafulou (Sn‒Zn), Kangma (Sn‒Zn), and Huile (Sn‒Zn). It is a non-ferrous metal ore field with one of the largest Sn polymetallic reserves in the world^9^. The Tongkeng Sn polymetallic deposit is located in the western part of the Dachang ore field and has the largest non-ferrous metal reserves in this field. The central part of the Tongkeng deposit is interspersed with granitic porphyry dikes (eastern dikes). Zircon U‒Pb dating of the eastern dikes gives an age of 91 ± 1 Ma (i.e., Late Cretaceous^40^). The No. 92, No. 91, veinlet zone, and large vein zone orebodies are located to the west of the eastern dikes, whereas the No. 96, No. 95, and No. 94 orebodies are located to the east of the eastern dikes (Fig. 1b).

The No. 92 orebody is characterised by laminar-banded, network-vein, nodular, and a small amount of interlayer vein mineralisation. The main ore minerals are sphalerite and FeS_2_; secondary ore minerals are cassiterite, arsenopyrite, and FeS; gangue minerals are primarily quartz, followed by calcite (CaCO_3_), tourmaline, and plagioclase.

The No. 91 orebody is primarily composed of laminar-banded and NE-trending jointed vein-like mineralisation. The main ore minerals are cassiterite, marmatite, arsenopyrite, and FeS, followed by FeS_2_. The main gangue minerals are quartz and tourmaline, followed by CaCO_3_ and potassium feldspar.

The orebody of the veinlet zone primarily consists of veinlet mineralisation with local laminar-banded mineralisation. The main ore minerals are marmatite, FeS_2_, and jamesonite, followed by cassiterite, arsenopyrite, FeS, and franckeite. The main gangue minerals are CaCO_3_, quartz, and tourmaline.

The orebody in the large vein zone is mineralised primarily by joint veins. The main ore minerals are marmatite, FeS_2_, jamesonite, and franckeite, followed by cassiterite and arsenopyrite.

## Results

### Mineralogical and geochemical signatures of hydrothermal vent sediments

**Figure 2:**
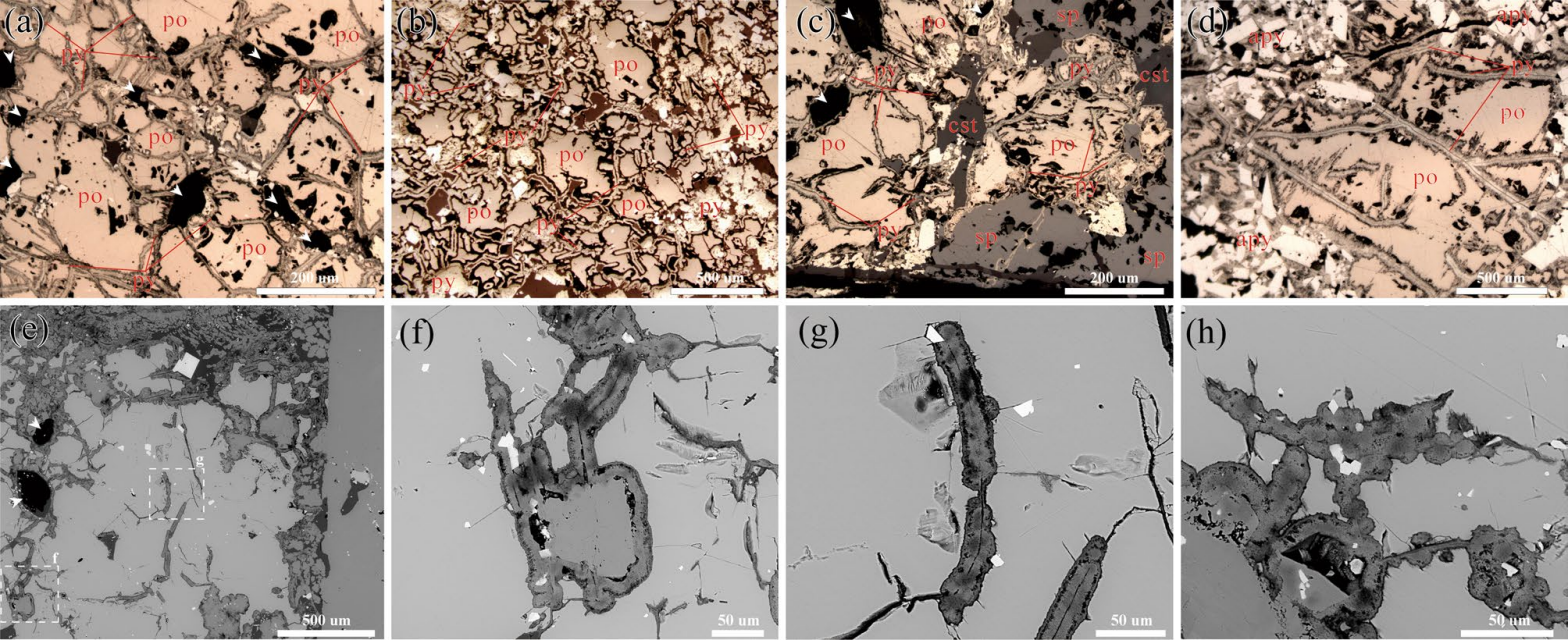
Microtextural characteristics of pyrite (FeS_2_) and related mineralogy. Tubular, beaded, and spherical SRB traces composed of FeS_2_ were distributed within pyrrhotite (FeS). (**a**) Micro-hydrothermal vents, filamentous bodies, and FeS. (**b**) Tubular and spherical bodies, and FeS. The FeS_2_-containing filamentous and tubular SRB in (**c**) and (**d**) were metasomatised with arsenopyrite, sphalerite, and cassiterite to form a residual structure. (**e**‒**h**) Backscattered electron (BSE) image of FeS_2_ and related mineralogy. po = pyrrhotite (FeS); py = pyrite (FeS_2_); apy = arsenopyrite; sp = sphalerite; cst = cassiterite. The white arrows in (**a**) and (**c**) indicate micro-hydrothermal vents.

**Figure 3:**
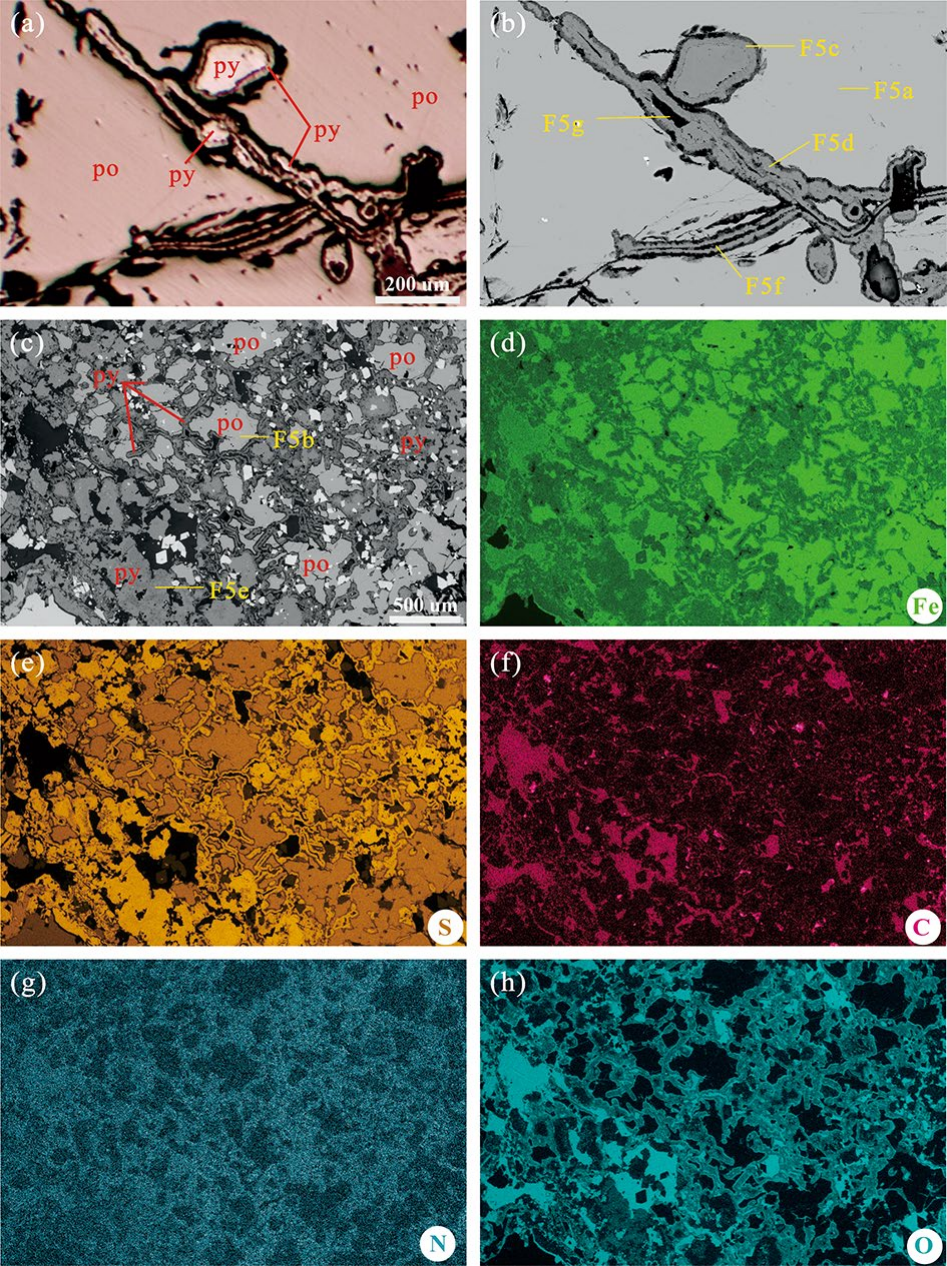
Plane polarised light photomicrograph, SEM‒EDX backscatter images, and EDX elemental maps of SRB traces. The analysed elements included S, Fe, C, N, and O. (**a**) Plane polarised light photomicrograph. (**b**) and (**c**) SEM‒EDX backscatter images. (**d**‒**h**) EDX elemental maps of Fe, S, C, N, and O, respectively.

Polarised microscopy and scanning electron microscopy of well-polished surfaces of the hydrothermal vent sediments showed that FeS_2_ formed tubular (Fig. 2a‒d,f‒g), beaded (Figs. 2h and 3a‒b), and coccoidal structures (Figs. 2e and 3c). Hydrothermal vent sediments also exhibited multi-stage mineralisation, in which the FeS_2_-containing filaments and tubes were metasomatised by minerals, including arsenopyrite, sphalerite, and cassiterite, to form remnants (Fig. 2c‒d). This multi-stage mineralisation indicates that FeS was formed in the earliest metallogenic process and that the formation environment of FeS_2_ was related to the Late Devonian mineralisation of submarine hydrothermal vent sediments. Numerous pores were filled with carbonate minerals in both samples (Fig. 2a and c), which may have constituted micro-hydrothermal vents. A similar phenomenon, known as the ‘ghosts’ of bacterial cells, has been observed in other submarine hydrothermal vent sediments as well as in modern vent settings and is believed to have resulted from bacterial iron accumulation on vestimentiferan tubes^41–43^.

Microscopic backscatter imaging and elemental analysis (Fig. 3) were performed in areas typical for the presence of SRB. An evident contrast was present in the mineral contents of FeS, FeS_2_, and non-metallic minerals in the backscattered electron images (Fig. 3b and c). In the EDX elemental images, areas of FeS showed high Fe and low S values, whereas those of FeS_2_ showed low Fe and high S values (Fig. 3d and e). C, N, and O were less present in FeS areas, all of which instead showed high values in areas of FeS_2_ and non-metallic minerals (Fig. 3f‒h).

### SRB occurrence and morphologies

**Figure 4:**
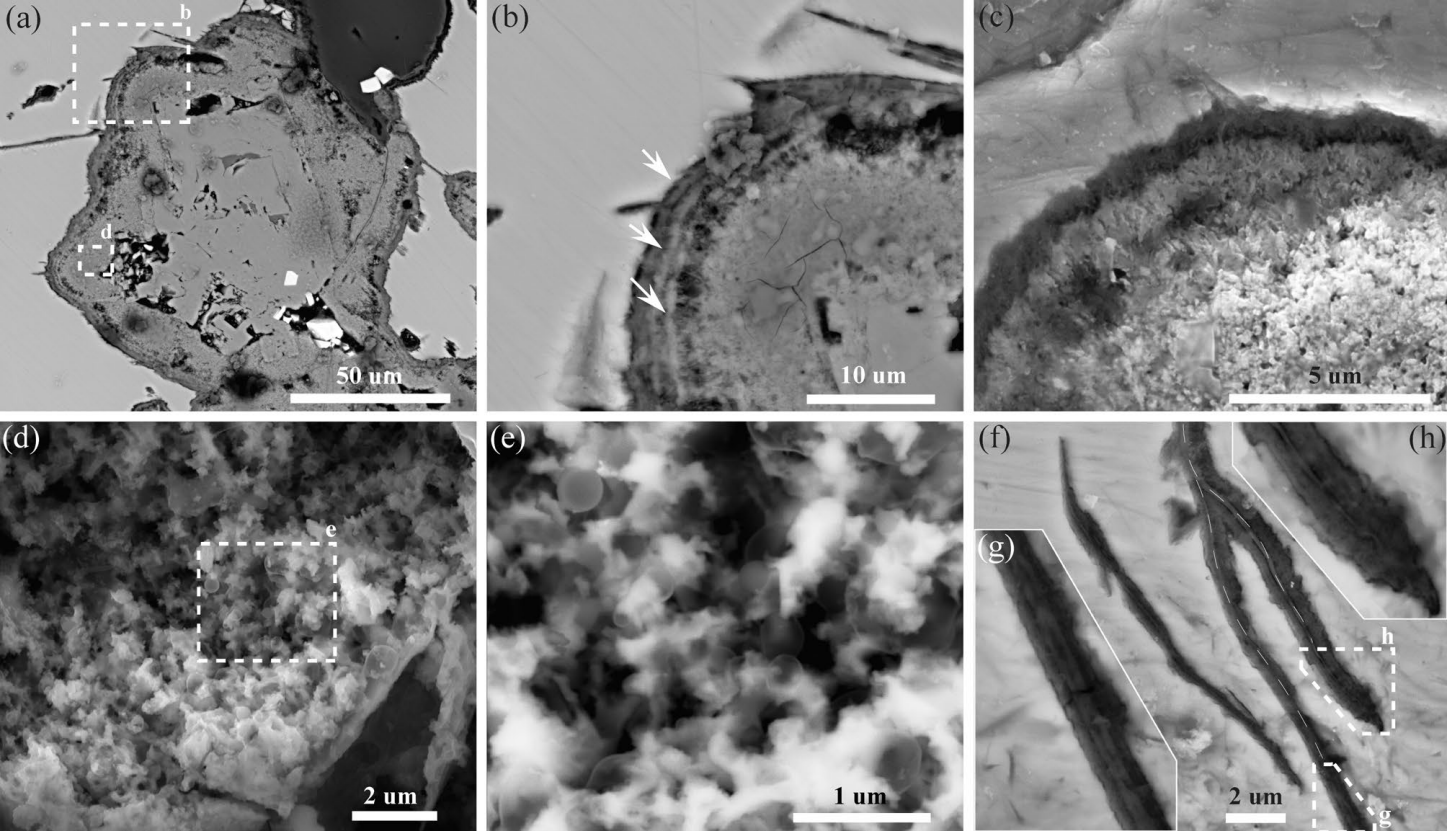
Electron images of SRB microstructure. (**a**) and (**b**) No nitric acid (HNO_3_)–etched FeS_2_. (**c**–**h**) 70% HNO_3_ etched FeS_2_. (**a**) Micro-hydrothermal vents. (**b**) Layered structures at the edges of the FeS_2_. (**c**) HNO_3_-etched layered structures of the FeS_2_ edges. (**d**) and (**e**) Backscattered electron image of spheroidal SRB fossils. (**f**) and (**h**) Backscattered electron image of filamentous SRB.

As observed using microscopy, FeS_2_ containing SRB was typically distributed within FeS, with miniature hydrothermal vents serving as the main channel for SRB dispersion. FeS_2_ exhibited various morphologies, including tubular (Fig. 2f and g), beaded (Fig. 2h), and coccoidal forms (Figs. 2 and 4a). Among these, a substantial portion of tubular FeS_2_ had relatively similar widths of approximately 25 μm (Fig. 2a‒e and g), whereas a small proportion of this type of FeS_2_ had narrower widths of approximately 1 μm (Fig. 4f‒h). Tubular FeS_2_ structures often connect multiple hydrothermal vents and occasionally display nodular structures at the edges (Fig. 2g and h). Some tubular FeS_2_ formations symmetrically develop hemispherical structures on both sides, with radii ranging from 10 to 25 μm, creating a beaded overall morphology (Fig. 2h). Additionally, some FeS_2_ existed as individual spherical particles, with diameters varying greatly from 20 to 50 μm (Fig. 2e). This variation in size may be attributed to spatial differences in the three-dimensional morphology of FeS_2_. Coccoidal and beaded FeS_2_ often exhibit distinctive layered structures at the edges (Fig. 4b).

In this study, a 70% nitric acid solution was used to etch the potential FeS_2_ regions containing SRB to characterise SRB microstructure. Etched interiors uncovered a substantial presence of spherical and filamentous residues, which were predominantly organic in nature due to the strong corrosive properties of concentrated nitric acid (Fig. 4c‒h). Moreover, in the backscattered electron images, these residues exhibited increased contrast in comparison to the FeS_2_ particles, further substantiating their association with SRB (Fig. 4c‒h). The embedding of spherical SRB fossils (ranging in size from 250 to 450 nm) within the FeS_2_ matrix indicates that these spheres were unadulterated by contaminants (Fig. 4d,e). Similar spherical microorganisms have also been found in modern hydrothermal vent environments from the eastern Manus Basin, where they have been observed at the layered periphery of FeS_2_, which aligns with the locations in which SRB were found in the Roman Ruins black smokers^44^. Additionally, filamentous SRB fossils exhibiting distinct branching patterns were preserved within a small number of tubular FeS_2_ structures after acid etching (Fig. 4f). The diameter of individual filaments averaged approximately 120 nm, and a pronounced curvature could be observed at the endpoints of the filamentous fossils, suggesting that they froze in a phase of outward growth (Fig. 4h). There were also instances in which multiple filamentous SRB fossils were preserved together (Fig. 4g).

### Raman spectroscopy

Because of being non-destructive, rapid, and convenient, Raman spectroscopy has been extensively used to identify valuable biological remains in sedimentary and metamorphic rocks^45–49^. The method has also been used as an effective tool for determining the microstructure of suspected biological specimens, potential microfossils, or other substances from various geological periods, especially when assessing their carbonaceous composition^50–52^.

**Figure 5:**
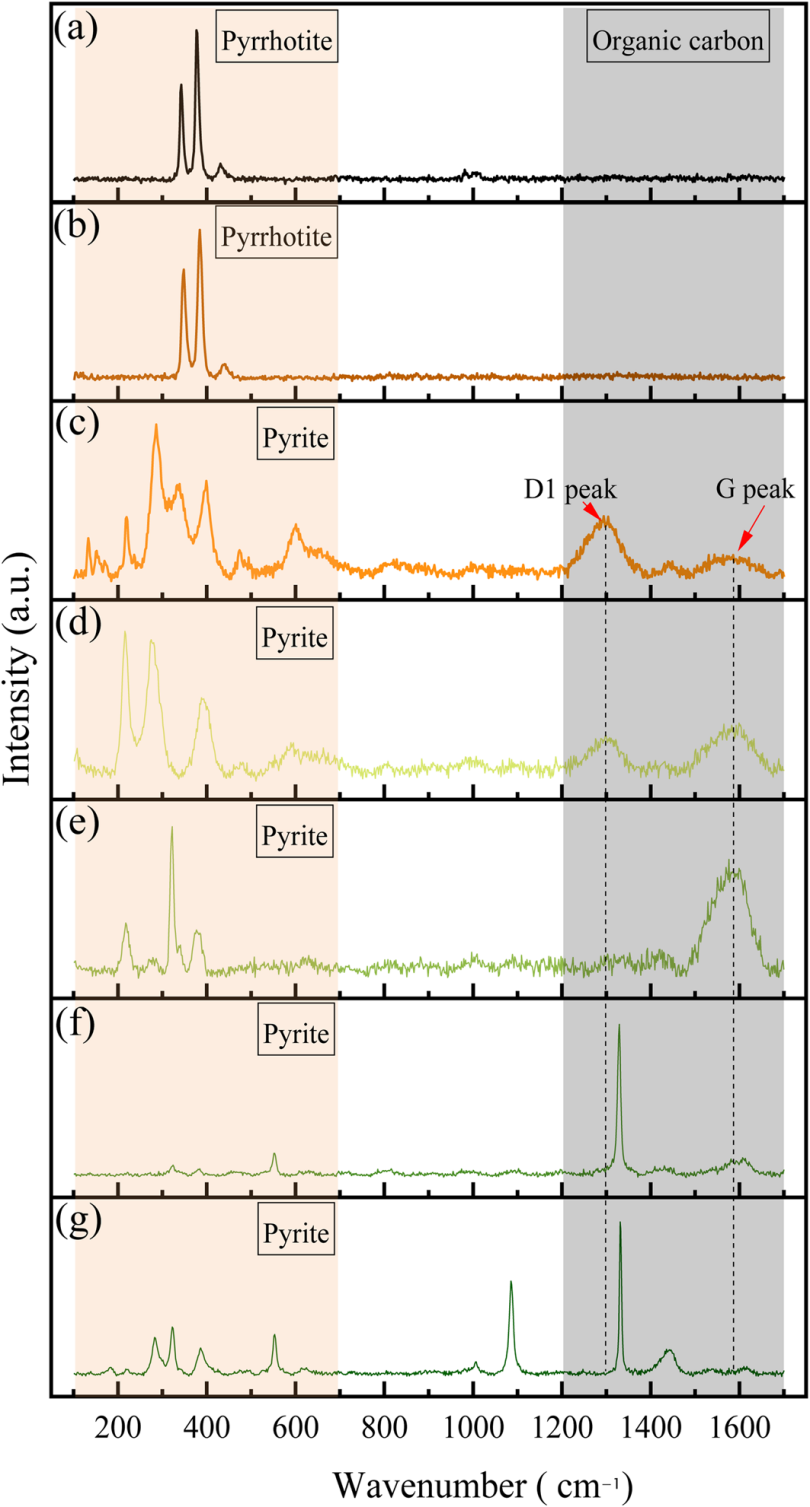
Representative results from Raman spectroscopy analyses on major minerals and putative microbial features. (**a** and **b**) Pyrrhotite. (**c**‒**g**) Pyrite. Measurement point locations are shown in Fig. 3b and c.

In this study, Raman spectroscopy analyses were performed on FeS, spheroidal FeS_2_ edges, tubular FeS_2_ walls, and the inner carbonate minerals of tubular FeS_2_ (Fig. 5; Supplementary information). The locations of the analysis points are shown in Fig. 3b and c. The laser Raman spectral characteristics of the different types of minerals clearly differed. Peaks of FeS and FeS_2_ primarily occurred in the range of 100–700 cm^−1^ (Fig. 5a and b), whereas for FeS_2_, several peaks were also observed in the range of 1000–1700 cm^−1^, including the D1 and G peaks of carbonaceous material located near 1300 cm^−1^ and 1590 cm^−1^, respectively (Fig. 5c‒g). Among these, the D1 peak is attributed to the presence of incorporated aromatic or benzene clusters, and the G peak is composed of the E2g2 mode of graphite or sp^2^ C=C stretching vibrations^53^. No discernible D2 peak was observed in the carbon materials associated with SRB, indicating a limited degree of graphitisation^54^. The absence of a D2 peak is directly influenced by the maximum environmental temperature experienced by the carbon material^51^. Intriguingly, the positions and morphologies of the D and G peaks in the Raman spectra of the carbon materials investigated in this study closely resembled the Raman signals documented in previous studies on kerogen^55,56^. In the case of these kerogens with relatively low maturity, the D2 peak was frequently insignificant or could not distinctively be separated from the G peak (Fig. 5d and e). This similarity supports the organic origin of the carbon signal detected in the FeS_2_ region using EDX analysis. These findings thus further corroborate the identification of the previously observed microstructures as bacterial microfossils^57^.

### Stable isotope composition

Our stable isotope analyses showed that the δ^56^Fe values of the six Fe-sulphide samples were dominated by weak positive excursions (Table 1). The mean δ^56^Fe value of FeS was 0.10‰, and that of FeS_2_ was 0.55‰. The δ^56^Fe values of FeS_2_ isolated from each sample were higher than those of the corresponding FeS. In addition, the δ^13^C_-PDB_ of organic C in the ore showed a strong negative anomaly, with a mean value of − 34.82‰, and the δ^13^C_-PDB_ of CaCO_3_ exhibited a weak negative excursion, with a mean value of − 0.40‰ (Table 1).

**Table 1:** Fe‒S‒C‒O stable isotope composition.

Sample	Composition	δ^56^Fe (‰)		δ^34^S (‰)		δ^13^C_-PDB_	δ^18^O_-PDB_
		Pyrrhotite	Pyrite	Pyrrhotite	Pyrite	(‰)	(‰)
TK-53-1	Fe-sulphides	0.03	0.11	− 4.49	− 4.17		
TK-53-2	Fe-sulphides	− 0.43	0.77	− 4.80	− 4.30		
TK-53-3	Fe-sulphides	0.71	0.78	− 4.91	− 4.82		
Mean		0.10	0.55	− 4.73	− 4.43		
TK-53-organic 1	Organic carbon					− 35.67	
TK-53-organic 2	Organic carbon					− 33.63	
TK-53-organic 3	Organic carbon					− 35.15	
Mean						− 34.82	
TK-53-carbon 1	Calcite					− 0.11	− 15.73
TK-53-carbon 2	Calcite					− 0.60	− 16.72
TK-53-carbon 3	Calcite					− 0.50	− 18.92
Mean						− 0.40	− 17.12

## Discussion

### Role of SRB in isotopic fractionation

The reaction between H_2_S generated by the activity of SRB and early-stage FeS to form FeS_2_ is a matter of debate^13^. Views on whether S is gained (Fe‒S bond is not broken) or Fe is lost (Fe‒S bond is broken) in the reaction process have been conflicting^58,59^. However, more recent studies reported that the process involves both loss of Fe and gain of S^60–62^. Our Fe-isotope analyses show that the δ^56^Fe values of FeS_2_ isolated from each sample were higher than those of the corresponding FeS (0.45‰ higher on average). We therefore speculate that some ^54^Fe isotopes were lost during the SRB-mediated conversion of FeS to FeS_2_, resulting in an increase in the proportion of ^56^Fe in the synthesised FeS_2_ compared with that of the original FeS.

The S-isotope composition of FeS and FeS_2_ is related to the environmental S-isotope composition. The supply of H_2_S in hydrothermal vents derives from both inorganic and organic processes^63–65^. Thermochemical sulphate reduction (TSR) occurs at high temperatures before the SO_4_^2−^-containing hydrothermal fluid is ejected from the seabed, resulting in S-isotope fractionation^66,67^. The generated H_2_S gas is enriched in light S isotopes, whereas the ejected hydrothermal SO_4_^2−^-rich solution is enriched in heavy S isotopes. Bacterial sulphate reduction (BSR) also causes S-isotope fractionation; the S-isotope composition of the resulting H_2_S gas depends on the S-isotope composition of SO_4_^2−^ in the environment and the extent of selective light S-isotope enrichment during cell-specific sulphate reduction^68,69^.

The S-isotope analysis showed that the δ^34^S values of FeS and FeS_2_ ranged from − 4.91 to − 4.17‰, with a mean of − 4.58‰ and a standard deviation of 0.28‰ (Table 1). We infer these abnormal negative ^34^S values to be related to TSR and BSR. The δ^34^S values of FeS_2_ isolated from the samples were higher than those of the corresponding FeS, which is probably related to the utilisation of SO_4_^2−^ with a high ^34^S content in SRB-mediated FeS_2_ synthesis. Researchers have analysed the S-isotope composition of barite (BaSO_4_) and various metal sulphides in other submarine hydrothermal vent systems^70^, showing that BaSO_4_ has the heaviest S-isotope composition. BaSO_4_ is precipitated by the combination of Ba^2+^ and SO_4_^2−^ in hydrothermal vents, and its S-isotope composition is similar to that of SO_4_^2−^ in the vent environment, indicating that SO_4_^2−^ has a relatively heavy S-isotope composition in vent systems. In addition, the strong negative anomaly of δ^13^C_-PDB_ reflected the selective absorption of light C isotopes by SRB.

### Precipitation of metallic minerals in hydrothermal vents

Submarine hydrothermal vent fluids are often rich in Fe^2+^, Pb^2+^, Zn^2+^, and other metal cations^2,71^. With drastic changes in the external physical and chemical environment, metal cations in hydrothermal fluids can easily combine with S, resulting in metal sulphide precipitation and accumulation^2^. Hydrogen sulphide (H_2_S) is an important factor that induces Fe ion precipitation; Fe ions can react with H_2_S to form FeS, as shown in Eq. (1)^13^:

1 $${\text{Fe}}^{{{2} + }} + {\text{ H}}_{{2}} {\text{S }} = {\text{ FeS }} + {\text{ 2H}}^{ + }$$

H_2_S can be formed in submarine hydrothermal vents by both inorganic and organic processes. SO_4_^2−^ pyrolysis in vent hydrothermal fluids can release H_2_S, which exhausts the vents^63,64^. The organic production of H_2_S in submarine hydrothermal vents is related to SRB, the primary producers of the vent ecosystem, which are both chemoautotrophic and organo-heterotrophic. H_2_ or organic matter can be used as an electron donor and SO_4_^2−^ as an electron acceptor to reduce SO_4_^2−^ to H_2_S and obtain energy in the reaction process. These reactions are given in Eqs. (2) and (3), respectively^72^:

2 $${\text{4H}}_{{2}} + {\text{ 2H}}^{ + } + {\text{ SO}}_{{4}}{^{{{{2} - }}}} = {\text{ H}}_{{2}} {\text{S }} + {\text{ 4H}}_{{2}} {\text{O}}$$

3 $${\text{2CH}}_{{2}} {\text{O }} + {\text{ SO}}_{{4}}{^{{{2} - }}} = {\text{ H}}_{{2}} {\text{S }} + {\text{ 2HCO}}_{{3}}{^{ - }}$$

H_2_S generated by organic and inorganic processes near hydrothermal vents rapidly combines with metal cations to form metal sulphides. Fe is first precipitated in the form of unstable mackinawite, greigite, or FeS; Zn and Pb form sphalerite (ZnFeS_2_), galena (PbS_2_), and other metal sulphides. The HCO_3_^−^ generated by the reaction in Eq. (3) easily combines with Ca^2+^ and/or Mg^2+^ ions in the ocean and is deposited in the form of CaCO_3_ or dolomite.

At present, two main FeS_2_ formation pathways are known: polysulfide (S_n_^2−^) Eq. (4) and H_2_S Eq. (5)^60,73^:

4 $${\text{FeS }} + {\text{ S}}_{n}{^{2 - }} = {\text{ FeS}}_{{2}} + {\text{ S}}_{n - 1}{^{2 - }}$$

5 $${\text{FeS }} + {\text{ H}}_{{2}} {\text{S }} = {\text{ FeS}}_{{2}} + {\text{ H}}_{{2}}$$

S_n_^2−^ and H_2_S can be produced in marine environments by inorganic processes or by processes involving microbes. In the S_n_^2−^ pathway, FeS_aq_ is attacked by nucleophilic polysulphides to form FeS_2_ (Eq. 4). In the H_2_S pathway, FeS_2_ is formed by electron transfer via the inner sphere complex between FeS and H_2_S (Eq. 5)^60,73^. The O_2_-deficient and sulphur-rich environment of the early Earth can satisfy both reactions, and the conversion of FeS to FeS_2_ is considered to be the key energy transfer reaction for the emergence of life^74,75^. SRB can efficiently synthesise S_n_^2−^ and H_2_S and promote FeS_2_ formation.

We infer that the precipitation of FeS is caused by Fe^2+^ and H_2_S reactions (a mixture of H_2_S generated by inorganic and organic processes) in hydrothermal vents, whereas the formation of tubular and spherical FeS_2_ is related to the presence and metabolic activities of SRB in the two massive sulphide ores within the Tongken Sn polymetallic deposit.

### SRB growth pattern

Microscopic observations show that SRB extended and expanded in peripheral FeS in the form of filaments, tubes, and spheroids (Fig. 2). As this is difficult to achieve in consolidated FeS, the most likely explanation is that precipitation of FeS and the activities of SRB took place concurrently. Specifically, as Fe^2+^ and H_2_S react to form FeS precipitates in hydrothermal vents, SRB migrate from the micro-hydrothermal vent to colonise the surrounding area and synthesise FeS_2_. Subsequently, both FeS and FeS_2_ precipitate with the co-occurrence of small amounts of SRB.

The EDX elemental maps revealed that the contents of C, N, and O in FeS were extremely low (Fig. 3f‒h). Furthermore, in FeS, no distinct peak was observed within the 1000–1700 cm^−1^ range, which is where organic matter peaks typically manifest in the laser Raman spectrum (Fig. 5a and b). Consequently, organic matter was nearly absent in FeS, suggesting minimal external organic matter input into the studied micro-hydrothermal vents. Therefore, the observed SRB may represent chemoautotrophic bacteria^76,77^, which rely on H_2_ supplied by the vent hydrothermal fluid as an electron donor and on SO_4_^2−^ as an electron acceptor to synthesise adenosine 5′-triphosphate in the cell to store energy needed for life, release H_2_S, and synthesise FeS_2_. Similar to the role of atmospheric CO_2_ in photosynthesis, CO_2_ derived from hydrothermal vent fluids can serve as the exclusive carbon source for cell synthesis during the growth of SRB^78^. The necessary N for SRB may come from N-containing ions such as NH_4_^+^, NO_3_^−^, or NO_2_^−^ in the vent hydrothermal fluids. These ions can be obtained via processes such as nitrification, dissimilatory NO_3_^−^ oxidation, or NO_2_^−^ reduction^79–81^.

**Figure 6:**
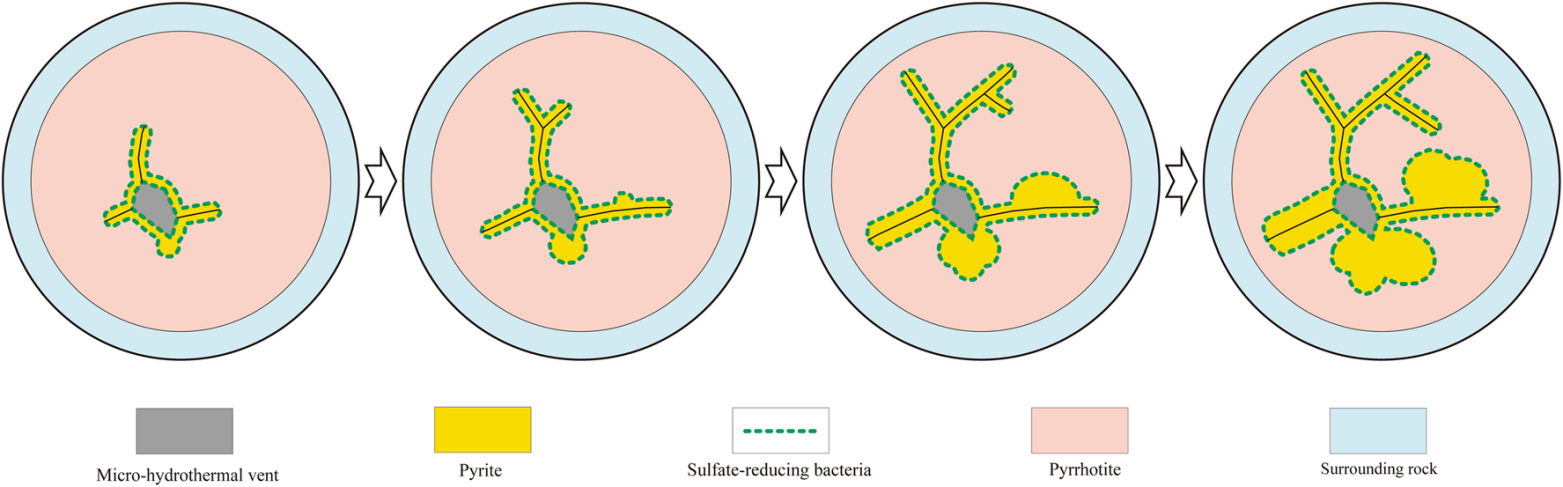
Sulphate-reducing bacteria growth modes. Sulphate-reducing bacteria (green dots) growing at a micro-hydrothermal vent (grey); chemoautotrophic SRB proliferate, branch, and expand around the micro-hydrothermal vent in tubular and spherical shapes. Yellow represents pyrite (FeS_2_), orange represents pyrrhotite (FeS), and blue represents seawater.

Based on the morphological data of SRB traces obtained using polarised light microscopy and SEM, combined with the EDX analysis, isotopic analysis, and Raman spectroscopy, we inferred the SRB-mediated FeS_2_ formation process in FeS under the theoretic framework of hydrothermal vent mineralisation. Microbes preserved in FeS_2_ are chemoautotrophic SRB growing in hydrothermal vents on the seabed, and hydrothermal fluids provide the materials and energy needed for their growth. Such bacteria live near micro-hydrothermal vents and rely on H_2_ as an electron donor and SO_4_^2−^ as an electron acceptor in the hydrothermal fluid to produce energy and H_2_S. H_2_S combines with unconsolidated FeS around SRB to form FeS_2_. After ore consolidation, these tubular, beaded, and spherical FeS_2_ structures are preserved within FeS, thus recording the morphological characteristics of SRB activity. SRB grow from micro-hydrothermal vents to their peripheries; tubular bodies may branch and thicken, sometimes spheroids may develop, and some spheroids may grow and proliferate directly from the micro-vents (Fig. 6). Tubular bodies are often interconnected between adjacent micro-vents (Fig. 2a–e). FeS_2_ synthesised by SRB via sulphate reduction presents the above microbial trace characteristics enclosed within the FeS matrix. Although H_2_S generated by microbial metabolic processes may promote the precipitation and mineralisation of galena, sphalerite, chalcopyrite, and other metal sulphides, no biomorphs of SRB have been found in these metal sulphides; this is worth further exploration in future studies.

## Conclusions

The genesis of the Tongkeng Sn polymetallic deposit is related to the mineralisation of Devonian submarine hydrothermal vent sediments. We investigated ores that contained both morphological and chemical evidence of SRB. Tubular, beaded, and spherical FeS_2_ structures were discovered in close proximity to micro-hydrothermal vents, displaying morphological characteristics typical of SRB flora. By analysing the elemental composition, employing Raman spectroscopy, and conducting isotopic analysis, we confirmed that the formation of FeS_2_ is a result of the metabolic activities of SRB, which involves reduction of SO_4_^2−^ and production of H_2_S. We also describe the presence of coccoidal and filamentous biomorphs, which we interpreted as fossil remains of SRB. The trace fossils of filamentous, tubular, and spherical FeS_2_ enclosed within FeS, along with their elemental and isotopic composition, provide a foundation for understanding microbial activities in sulphur-rich anoxic environments. Moreover, our study also presents a novel observational method for studying submarine vent hydrothermal ecosystems and exploring the potential for life in extreme environments.

## Materials and methods

### Sample collection

We performed a systematic geological survey and sampling work in the Tongkeng Sn polymetallic deposit. A total of 70 massive sulphide ore samples were collected and transformed into polished thin sections for microscopic identification and chemical composition analysis. Using a polarising microscope, we found SRB trace fossils in two samples. These two samples were numbered TK-51 and TK-53 and were taken from the No. 92 ore body within the ore-bearing Liujiang Formation (D3L).

### SEM–EDX

Our SEM‒EDX analysis was performed using an FEI Quanta 650 FEG scanning electron microscope equipped with an EDAX EDX detector under low vacuum. Backscattered images were captured under the following microscope setup: 15‒20 kV accelerating voltage, 5.5 spot size, 10.2 mm working distance, and 100 Pa chamber pressure. Elemental maps were collected with a count rate exceeding 10,000 cps and a dead-time of approximately 15%.

### Raman microspectroscopy

Micro-Raman spectra were collected using an inVia Qontor confocal Raman instrument (Renishaw Plc, UK). The laser beam was focused on the sample through a 50× objective lens with 532 nm radiation provided by a solid-state laser. The laser power was set at 10 mW on the sample surface. Baseline correction and spectral peak fitting of the Raman spectra of all specimens were performed using Origin Pro-2021 (Learning Edition) and PeakFit (v. 4.12).

### Isotope ratio mass spectroscopy

Three sub-samples for stable isotope analysis were obtained from sample TK-53; phases analysed included FeS, FeS_2_, CaCO_3_, and organic C, and the elements analysed were Fe, S, C, and O. The three ore samples were crushed and sieved until the particle diameters were < 0.425 mm; FeS was first adsorbed with a magnet and then screened with a binocular microscope (20× magnification) to obtain FeS without impurities. FeS_2_, CaCO_3_, and non-metallic particles with high carbonaceous content were selected from non-magnetic mineral particles by screening using a binocular microscope.

The three groups of FeS and FeS_2_ samples were powdered for Fe and S stable isotopic analysis. Fe stable isotope analysis of FeS and FeS_2_ was performed by Guangzhou ALS Chemex (China). S stable isotope analyses were performed in the stable isotope analysis laboratory of the Kunming University of Science and Technology (China). S in FeS and FeS_2_ was converted to SO_2_ by a high-temperature combustion method using an elemental analyser (Vario isotope cube, Elementar, Germany), which was then transferred into a gas-phase isotope ratio mass spectrometer (Isoprime-100, Elementar) to analyse its S-isotope composition.

The C and O isotopic components of CaCO_3_ were converted to CO_2_ by the phosphoric acid method using Isoprime multiflow equipment in the stable isotope analysis laboratory of Kunming University of Science and Technology, which was then transferred into a gas-phase isotope ratio mass spectrometer (Isoprime-100) to analyse its C and O stable isotope composition.

Non-metallic particles with a high C content were soaked in dilute hydrochloric acid, washed, and dried twice to remove carbonate minerals. In the stable isotope analysis laboratory of the Kunming University of Science and Technology, the organic C present in ores was converted into CO_2_ using a high-temperature combustion method, which was then transferred into a gas isotope ratio mass spectrometer (Isoprime-100) to analyse its C stable isotope composition.

## Supplementary Information

Supplementary Information.

## Data Availability

All data that support the findings of this study are available in the paper. Source data are provided with this paper.
